# Effect of Dentin Bonding Agents, Various Resin Composites and Curing Modes on Bond Strength to Human Dentin

**DOI:** 10.3390/ma12203395

**Published:** 2019-10-17

**Authors:** Rene Steiner, Daniel Edelhoff, Bogna Stawarczyk, Herbert Dumfahrt, Isabel Lente

**Affiliations:** 1 Medical University of Innsbruck, University Hospital for Dental Prosthetic and Restorative Dentistry, Anichstraße 35, 6020 Innsbruck, Austria; rene.steiner@tirol-kliniken.at (R.S.); herbert.dumfahrt@gmx.at (H.D.); 2 Department of Prosthetic Dentistry, University Hospital, LMU Munich, Goethestrasse 70, 80336 Munich, Germany; daniel.edelhoff@med.uni-muenchen.de (D.E.); Isabel.metz@med.uni-muenchen.de (I.L.)

**Keywords:** direct restoration, dentin bonding, adhesive system, resin composite, curing mode, push-out bond strengths

## Abstract

This study investigated the influence of several dentin bonding agents, resin composites and curing modes on push-out bond strength to human dentin. 360 extracted caries-free third molars were prepared, cut into slices, embedded in epoxy resin and perforated centrally. One half of the specimens (180) were treated by using one-step adhesive systems and the other half (180) with multi-step adhesive systems. Subsequently, the cavities were filled with either universal, flowable or bulk-fill resin composite according to the manufactures’ product line and cured with either turbo or soft start program. After storage the push-out test was performed. The data was analyzed using Kolmogorov-Smirnov, three- and one-way ANOVA followed by the Scheffé post-hoc test, unpaired two-sample t-test (*p* < 0.05). The strongest influence on push-out bond strength was exerted by the resin composite type (partial eta squared η_P_^2^ = 0.505, *p* < 0.001), followed by the adhesive system (η_P_^2^ = 0.138, *p* < 0.001), while the choice of the curing intensity was not significant (*p* = 0.465). The effect of the binary or ternary combinations of the three parameters was significant for the combinations resin composite type coupled adhesive system (η_P_^2^ = 0.054, *p* < 0.001), only. The flowable resin composites showed predominantly mixed, while the universal and bulk-fill resin composite showed adhesive failure types. Cohesive failure types were not observed in any group. Multi-step adhesive systems are preferable to one-step adhesive systems due to their higher bond strength to dentin. Flowable resin composites showed the highest bond strength and should become more important as restoration material especially in cavity lining. The use of a soft start modus for polymerization of resin composites does not enhance the bond strength to dentin.

## 1. Introduction

Clinical success and long-term survivability depend on multiple factors. Besides the operator themselves and the physical conditions, several parameters can affect integration by influencing the quality of the interface and the bond strength between the resin composite and the hard tooth tissue [1,2]. During polymerization, the stress at the interface is regulated by factors such as the proportion between bonded and non-bonded surface area (c-factor), the bond strength of the adhesive, the module of elasticity of the restoration material, the shrinkage in volume, the mode of light curing and the degree of conversion [3,4,5,6]. Disintegration of the interface provokes a leakage between the resin composite material and the tooth [7,8]. Postoperative hypersensitivity, marginal discoloration, bacterial invasion and secondary caries can occur [9,10]. Therefore, the choice of an adhesive system, a resin composite and a curing mode can have an impact on the clinical success of a direct resin composite restoration and has to be made carefully. 

Nowadays, strategies that facilitate the operator in reducing the time of treatment by simplifying the work process are becoming increasingly attractive. Timesaving systems can be found for almost every treatment step. Therefore, various systems are available for adhesive bonding, restorative materials and curing modes. Adhesive bonding systems can be divided into multi-step and one-step systems. For multi-step etch-and-rinse adhesive systems it is necessary to pretreat the hard tooth tissue with 37% phosphoric acid gel for 15 to 30 s, while a pretreatment by using a one-step adhesive system is not necessary [11]. In addition, multi-step systems are more time-consuming and sensitive to technical errors because of multiple application steps [12]. Resin composite material can be classified into groups according to the material viscosity: flowable, universal and bulk-fill resin composites. Since flowable and universal composites are well established on the market the third group of bulk-fill composites is relatively young. Bulk-fill resin composites show higher polymerization depth and reduced shrinkage stress compared to conventional resin composites [8,13]. These characteristics allow the operator to layer increments of up to 4 mm in a single step, which reduces treatment time as well [14,15]. Differing curing modes are available, depending on the polymerization light. Conventional polymerization lights are used for 15 to 20 s per increment [16,17]. Specialized “soft-start” programs start with a reduced intensity of 600 mW/cm^2^ for five seconds, followed by 1200 mW/cm² for an additional 15 s. Special lights with high intensity (<2000 mW/cm^2^) can be used in “turbo programs” for a reduced polymerization time of 5 s, which saves time, but increases the risk of pulp irritation caused by heat development [18,19,20]. The huge variety of alternatives and system options to treat a cavity with a direct resin composite restoration launched on the market poses a challenge for the operator to make a profound choice. Therefore, it is necessary to investigate the impact of different systems on the clinical success of resin composite restorations. In the literature, it has been shown that the push-out test is an adequate and effectively method to test the bond strength [21,22,23,24,25]. Consequently, this study uses the push-out test to investigate the impact of several factors on the interface between resin composite and hard tooth tissue. The aim of this study is to examine the influence of different adhesive systems (one-step, multi-step), resin composites (flowable, universal, bulk-fill) and curing modes (soft-start, turbo). The following hypotheses were tested:

1. One-step adhesive systems show comparable bond strengths to multi-step adhesive systems.
2. Flowable resin composites, universal resin composites and bulk-fill resin composites produce almost equal bond strengths.
3. The curing program has no impact on bond strengths.

## 2. Materials and Methods

**Figure 1:**
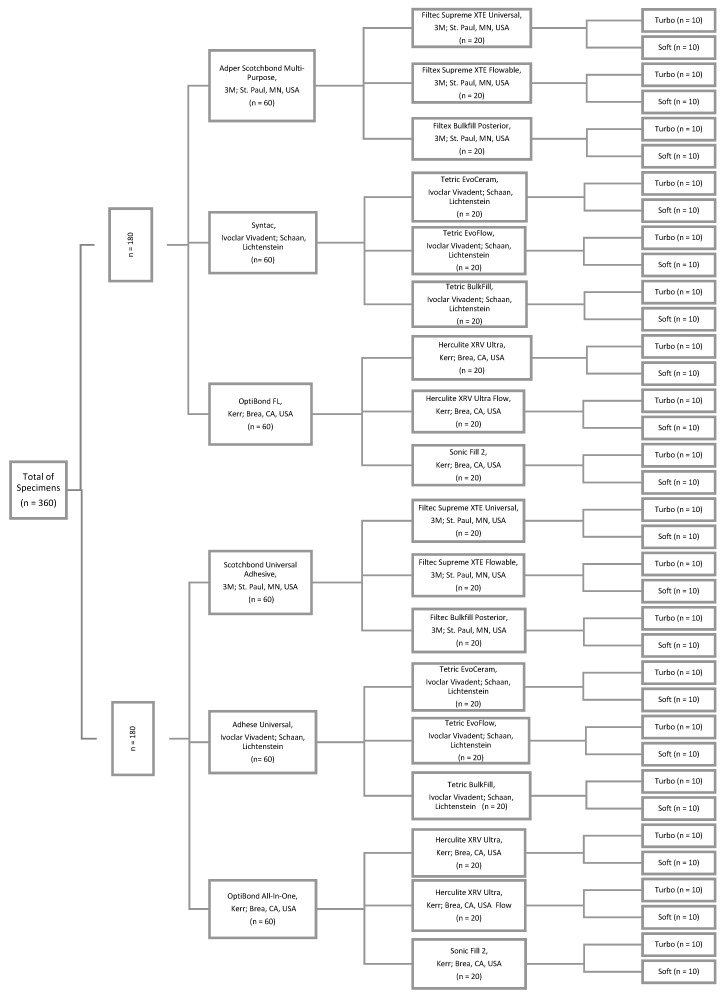
Study design.

**Figure 2:**
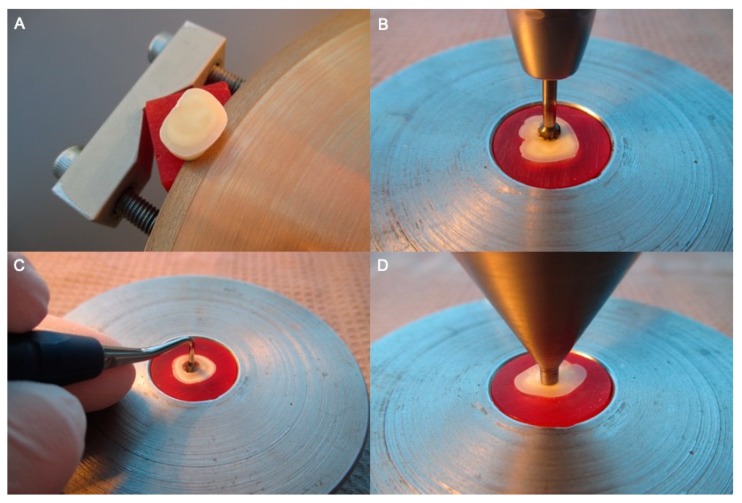
Specimens preparation: (**A**) Cutting process; (**B**) Drilling procedure; (**C**) Manual cavity filling; and (**D**) Push-out test procedure.

In this study, 360 extracted caries-free third molar were used (Figure 1). For disinfection, the teeth were submerged in 1% chloramine-T solution (CAS: 149358-73-6) for one week and subsequently stored in isotonic saline solution at 4 °C for a maximum of six months. Using a diamond disk at low speed under water irrigation (Isomet 1000, Buehler; Lake Bluff, IL, USA), the teeth were cut into slices of 4 ± 0.1 mm thickness (Figure 2A,B). Subsequently, the tooth slices were embedded in epoxy resin and a central perforation of 4 ± 0.1 mm in diameter was drilled using a carbide bure (ISO No. 500 104 001 251 040) (Fräsgerät S3-Master, Schick, Schemmerhofen, Germany). The specimens were cleaned from grinding debris using an Ethylendiamintetraacetat (EDTA) containing cleaning agent (Tubulicid blue, Dental Therapeutics AB, Saltsjö-Boo, Sweden) and distilled water. 

The total number of specimens was divided randomly into two groups (“One-Step-System”, “Multi-Step-System”) (two groups with n = 180 each). Figure 1 illustrates the study design with the manufacturer, city and country information of the materials used. The 180 specimens of the “Multi-Step-System” group were pretreated by applying 37% phosphoric acid gel (3M, St Paul, MN, USA) for 15 s, followed by rinsing with distilled water for one minute and drying with compressed water- and oil-free air. The 180 specimens of the “One-Step-System” group had no pre-treatment. Subsequently, the specimens of each group were divided into three sub-groups, according to the one-step/multi-step adhesive systems (“Scotchbond Universal Adhesive”, “Adhese Universal”, “OptiBond All-In-One”, “Adper Scotchbond Multi-Purpose”, “Syntac”, “OptiBond FL”) (six groups with n = 60 each). The adhesive systems were applied following the manufacturers’ instructions. The cavities were filled with resin composite material, also strictly following the manufacturers’ instructions. 20 specimens of each sub-group were filled with either universal, flowable or bulkfill resin composite of the manufacturers’ product line (Figure 2C). (“Filtec Supreme XTE Universal”, “Tetric EvoCeram”, “Herculite XRV Ultra”, “Filtec Supreme XTE Flowable”, “Tetric EvoFlow”, “Herculite XRV Ultra Flow”, “Filtec Bulk Fill Posterior”, “Tetric Bulk Fill”, “Sonic Fill 2”) (18 groups with n = 20 each). For light curing, an Light Emitting Diode (LED) curing light (Bluephase 20i Turbo Program, Ivoclar Vivadent; Schaan, Liechtenstein) was used while conducting two different modalities. The resin composite of ten specimens of each resin composite group were cured using the “Turbo Program” at 2000 mW/cm^2^ for five seconds, the other half (n = 10) were cured using the “Soft-Start Program” with 600 mW/cm^2^ for five seconds increasing to 1200 mW/cm^2^ for further 15 s (36 groups with n = 10 each). Excessive resin composite material was removed leveling the surface by grinding and polishing. Afterwards, all specimens were stored in isotonic saline solution for 24 h. 

The push-out tests were performed using a universal testing machine (Zwick Z010; Zwick-Roell; Ulm, Germany). Therefore, a cylindrical stainless punch with 3.4 mm in diameter was positioned creating a plane parallel contact to the resin composite surface (Figure 2D). Compressive load was applied moving downwards to a crosshead speed of 1 mm/minute until the resin composite detached off the slice-specimens. Push-out bond strength was calculated to Megapascals (MPa). Therefore, the load at failure (in Newtons) was divided by the bonded surface area (in mm^2^). The lateral bonded surface area (S) was calculated using the formula:

S = d × π × h = 4 mm × π × 4 mm ≈ 50.27 ± 1.39 mm^2^ (d: diameter of the cylinder, h: thickness of the slice).

The failure types were determined by stereomicroscope (BX 51M, Olympus, Tokyo, Japan) with 50× magnification and recorded as adhesive, cohesive or mixed. An adhesive failure type is defined by there being no remaining resin composite on the dentin wall, whereas the dentin wall is completely covered with resin composite in a cohesive failure type. As its name implies, the mixed failure type is characterized by exposed and covered dentin areas.

### Statistical Evaluation

The measured data were analyzed using descriptive statistics such as mean and standard deviation. Normality of data distribution was tested using the Kolmogorov-Smirnov. Three- and one-way ANOVA followed by the Scheffé post-hoc test were computed to determine the significant differences among the test parameters. The impact of adhesive system or the impact of polymerization type was calculated using an unpaired two-sample t-test. Relative frequencies of failure types were provided. A Chi2 test was used to detect differences in frequencies of failure types in different groups. The statistical tests were performed with SPSS Version 25.0 (SPSS INC, Chicago, IL, USA, *p* < 0.05).

## 3. Results

The highest influence on push-out bond strength was exerted by the resin composite type (partial eta squared η**_P_^2^** = 0.505, *p* < 0.001), followed by the adhesive system (η**_P_^2^** = 0.138, *p* < 0.001), while the choice of the curing intensity was not significant (*p* = 0.465). η**_P_**² stands for partial eta-squared and is a statistical parameter that measures the effect size, whereas the interpretation of the *p*-value alone is not sufficient enough to detect the influence of a factor [26]. The effect of the binary or ternary combinations of the three parameters was significant for the combinations resin composite type coupled adhesive system, only (η**_P_^2^** = 0.054, *p* < 0.001). 86% of all tested groups showed no violation of the normal distribution; therefore, the measured data were analyzed parametrically (Table 1).

Within the universal resin composite combined with one-step adhesive system and soft curing intensity, no impact of the used material was found (*p* = 0.060), while within the group polymerized by using turbo curing intensity, Tetric EvoCeram showed significantly lower push-out bond strength than Herculite XRV Ultra (*p* = 0.025). Within resin composite combined with multi-step adhesive systems, Tetric EvoCeram showed significantly lower bond strength compared to Filtec Supreme XTE Universal and Herculite XRV Ultra, regardless of the curing intensity.

Within the flowable resin composites Tetric EvoFlow showed significantly lower bond strength compared to Filtec Supreme XTE Flowable and Herculite XRV Ultra Flow, regardless of the adhesive system and curing intensity (*p* < 0.001).

Among the bulk-fill resin composites combined with one-step adhesive systems, the lowest results were observed for Sonic Fill 2 followed by Tetric Bulk-Fill (*p* = 0.002–0.018), regardless of the curing intensity. Among multi-step adhesive systems, Tetric Bulk-Fill showed the lowest and Filtec Bulk-Fill Posterior the highest bond strength values (*p* < 0.001).

For all resin composite types (universal/flowable/bulk-fill), the use of a multi-step adhesive system showed significantly higher bond strength than the one-step adhesive system (*p* < 0.001). However, the Tetric products were an exception. Within universal and bulk-fill resin composites no impact of adhesive system was measured (*p* = 0.515). Within flowable resin composite specimens bonded with TetricEvoFlow in combination with one-step adhesive system showed significantly higher bond strength than specimens bonded using multi-step ones (*p* < 0.001).

The flowable resin composites showed predominantly mixed, while the universal and bulk-fill resin composite showed adhesive failure types. Cohesive failure types were not observed in any group. The detailed distribution of the fracture types is presented in Table 2.

## 4. Discussion

The first hypothesis (one-step adhesive systems show comparable bond strengths to multi-step adhesive systems) was rejected because the use of multi-step adhesive systems showed significantly higher bond strength than one-step adhesive systems, except for the Syntac adhesive system. In the literature, there has been controversy in discussions of the characteristics of one-step and multi-step adhesive systems [27]. However, the main disadvantage of one-step self-etch adhesives is related to their excessive hydrophilicity, which influences the adhesive layer to be more prone to attract water from the intrinsically moist substrate [28]. Due to this increased water affinity, these one-step adhesives have been reported to act as semi-permeable membranes, even after polymerization, allowing water movement from the substrate throughout the adhesive layer [29]. Such permeability of the adhesive layer seems to contribute the hydrolysis of resin polymers and the consequent degradation of the tooth–resin bond over time [30].

The finding that the Syntac adhesive system, the only four-step system on the market with an additional priming step, showed lower mean shear bond strengths than the corresponding one-step system Adhese Universal was an exception. Syntac is an acetone-based, hydroxyethylmethacrylate (HEMA)-free and maleic acid containing adhesive system. Maleic acid, contained in the Syntac Primer, has a conditioning effect on the dentin surface. In the present study phosphoric acid was applied as conditioning agent. Maleic acid causes decalcification of hydroxylapatite [31]; therefore, it could be possible that the dentin was over-etched, which could have led to a reduction of the morphology. The fact that prolonged etching times on dentin may reduce the bond strength is well known [32]. However, it is remarkable that in each subgroup, Syntac revealed the lowest mean bond strength values. Another explanation could be that Syntac is a HEMA-free adhesive system. HEMA (2-Hydroxyethylmethacrylate), a hydrophilic monomer, is present in dental adhesive systems extensively since its molecular weight is rather low. It acts as a co-solvent and facilitates the blending of hydrophobic and hydrophilic ingredients into a single homogeneous blend. In addition, it assists the infiltration capacity into the demineralized dentin surface, which leads to an increase in bond strengths [33,34,35]. Zanchi et al. [36] compared HEMA-free and HEMA-containing adhesive systems and implied that the second were characterized by higher bond strength to dentin. Van Meerbeek et al. [37] came to the conclusion that HEMA-free adhesives are prone to phase-separation, which may account for their lower bonding effectiveness. A reason of introducing HEMA-free adhesive systems is that HEMA has been associated with allergic reactions extensively and is a trigger for inducing pulp apoptotic cell death when diffusing into dentinal tubules [36,37,38,39]. Furthermore, the Syntac adhesive differs from other adhesive systems in its solvent. While ethanol is the most commonly used solvent in adhesive systems, Syntac’s solvent is acetone. Acetone-based systems evaporate more water than ethanol-/water-based systems, and they are more sensitive to air-drying, as they cannot re-expand the shrunken collagen fibrils [12,40]. Ethanol-/water-based systems are less moisture-sensitive, and are effective in re-expanding collagen matrix, so they attain higher bond strengths in dried dentin [40,41]. In summary, it has been shown in this study that the acetone-based, HEMA-free and maleic acid containing adhesive system Syntac, created lower bond strength values than the other investigated adhesive systems.

The second hypothesis (flowable resin composites, universal resin composites and bulk-fill resin composites produce almost equal bond strengths) was also rejected because flowable resin composites showed higher mean push-out bond strength values than universal or bulk-fill resin composites. Flowable composites are characterized by a low filler content, whereby a homogeneous adaptation to the dentine surface is possible. Therefore, flowable composites are commonly used in lining, improving cavity adaptation and marginal sealing, increasing the microtensile bond strength [42,43] and reducing the polymerization shrinkage stress [44]. Furthermore, flowable composites are characterized by a lower elastic modulus than universal and bulk-fill composites [44]. Although bulk-fill resin composites are characterized by lower polymerization shrinkage due to stress reducing monomers, their higher elastic modulus may cause a reduction in bond strength to dentin. Actually, SonicFill, a single-step bulk-fill composite that comes with a special hand piece, which decreases the viscosity of composite upon activation of sonic energy [45,46], did not convince with its bond strength values. 

The third hypothesis (the curing program has no impact on bond strengths) was confirmed, because the mean push-out bond strengths after polymerization with the “Turbo Program” and the “Soft-start Program” did not differ statistically from one another. Although it has been described in the literature that polymerization under high intensity causes a higher shrinkage stress [17], which is reflected in the loss of bond between resin composite and dentin [47], the higher conversion of degree during the polymerization may compensate for this [17].

As a limitation of the study, it can be noted that determining the failure type the cohesion of the resin composite itself has been ignored. In other words, a mixed failure type can be caused by a high bond strength between resin composite and dentin or by a weak cohesion of the resin composite material. The comparison between the failure type and the material’s cohesion would be useful for a better interpretation of the fracture type.

It is suggested that future studies include significant clinical factors like mastication strain, change of temperature and storage in water or physiologic saline for weeks and months. Specimens would have to pass a thermocycling process and a chewing simulation prior and after the bond strength tests. This would be adequate for evaluating the in vitro longevity of the resin composite’s adhesion to dentin. 

## 5. Conclusions 

Within the limitations of the present study, the following conclusions can be drawn:

1. Multi-step adhesive systems are preferable to one-step adhesive systems due to their higher bond strength to dentin.
2. Flowable resin composites showed the highest bond strength values to dentin and should become more important as restoration material especially in cavity lining.
3. The use of a shrinkage-reducing soft-start mode for the polymerization of resin composites does not enhance the bond strength to dentin.
4. Acetone-based, HEMA-free and maleic acid-containing adhesives should be avoided due to their lower bond strength with dentin.

## Figures and Tables

**Table 1:** Push-out bond strength (MPa) and standard deviations (±SD) for each tested group separately.

Composite Material		One-Step Self-Etch Adhesive System	Multi-Step Etch-and-Rinse Adhesive System
Universal	Filtec Supreme XTE Universal	13.11 ± 5.04 (soft) ^a,A,I,y^ 13.04 ± 4.20 (turbo) ^a,AB,I,y^	18.44 ± 2.00 (soft) ^a,B,I,z^ 18.42 ± 2.23 (turbo) ^a,B,I,z^
	Tetric EvoCeram	11.90 ± 3.08 (soft) ^a,A,I,z^ 10.26 ± 2.91 (turbo) ^a,A,I,z^	11.23 ± 1.49 (soft) ^a,A,I,z^ 10.75 ± 3.12 (turbo) ^a,A,I,z^
	Herculite XRV Ultra	15.77 ± 1.67 (soft) ^b,A,I,y^ 15.27 ± 4.34 (turbo) ^a,B,I,y^	18.64 ± 1.72 (soft) ^a,B,I,z^ 18.86 ± 4.50 (turbo) ^a,B,I,z^
Flowable	Filtec Supreme XTE Flowable	22.91 ± 1.99 (soft) ^b,B,I,y^ 21.81 ± 2.16 (turbo) ^c,B,I,y^	25.54 ± 2.80 (soft) ^c,B,I,z^ 25.80 ± 2.81 (turbo)^c,B,I,z^
	Tetric EvoFlow	18.04 ± 2.51 (soft) ^b,A,I,z^ 17.51 ± 1.98 (turbo) ^b,A,I,z^	11.71 ± 1.59 (soft) ^a,A,I,y^ 10.90 ± 4.38 (turbo) ^a,A,I,y^
	Herculite XRV Ultra Flow	23.20 ± 2.39 (soft) ^c,B,I,y^ 22.69 ± 2.72 (turbo) ^b,B,I,y^	27.50 ± 2.58 (soft) ^b,B,I,z^ 27.18 ± 2.21 (turbo) ^b,B,I,z^
Bulk-Fill	Filtec Bulk-Fill Posterior	16.69 ± 4.48 (soft) ^a,B,I,y^ 17.29 ± 4.78 (turbo) ^b,B,I,y^	21.76 ± 2.84 (soft) ^b,C,I,z^ 22.33 ± 2.57 (turbo) ^b,C,I,z^
	Tetric Bulk-Fill	12.84 ± 2.43 (soft) ^a,A,I,z^ 12.07 ± 2.23 (turbo)^a,A,I,z^	12.23 ± 3.51 (soft) ^a,A,I,z^ 11.63 ± 1.71 (turbo) ^a,A,I,z^
	Sonic Fill 2	11.81 ± 4.09 (soft) ^a,A,I,y^ 12.73 ± 2.01 (turbo) ^a,A,I,y^	18.31 ± 3.08 (soft) ^a,B,I,z^ 18.92 ± 2.39 (turbo) ^a,B,I,z^

^abc^ indicated significant differences between the different material composite types within materials of one manufacturer and one curing type; ^ABC^ indicated significant differences between the different materials within one material composite type and one curing type; ^I,II^ indicated significant differences between the curing types within one material composite type and one manufacturer; ^xyz^ indicated significant differences between the adhesive types within materials of one manufacturer and one curing type.

**Table 2:** Relative frequencies with 95% confidence intervals [%(95%CI)] of adhesive fracture types for each tested group separately.

Composite Material		One-Step Self-Etch Adhesive System	Multi-Step Etch-and-Rinse Adhesive System
Universal	Filtec Supreme XTE Universal	80 (43;98) (soft) 70 (33;94) (turbo)	60 (25;88) (soft) 70 (33;94) (turbo)
	Tetric EvoCeram	70 (33;94) (soft) 80 (43;98) (turbo)	100 (68;100) (soft) 100 (68;100) (turbo)
	Herculite XRV Ultra	70 (33;94) (soft) 60 (25;88) (turbo)	70 (33;94) (soft) 50 (17;82) (turbo)
Flowable	Filtec Supreme XTE Flowable	30 (5;66) (soft) 40 (11;74) (turbo)	20 (1;56) (soft) 20 (1;56) (turbo)
	Tetric EvoFlow	30 (5;66) (soft) 40 (11;74) (turbo)	80 (43;98) (soft) 70 (33;94) (turbo)
	Herculite XRV Ultra Flow	20 (1;56) (soft) 30 (5;66) (turbo)	10 (0;45) (soft) 20 (1;56) (turbo)
Bulk-Fill	Filtec Bulk-Fill Posterior	50 (17;82) (soft) 40 (11;74) (turbo)	40 (11;74) (soft) 30 (5;66) (turbo)
	Tetric Bulk-Fill	40 (11;74) (soft) 50 (17;82) (turbo)	90 (54;100) (soft) 100 (68;100) (turbo)
	Sonic Fill 2	80 (43;98) (soft) 90 (54;100) (turbo)	70 (33;94) (soft) 80 (43;98) (turbo)
